# A cluster randomized trial evaluating a teachable moment communication process for tobacco cessation support

**DOI:** 10.1186/s12875-021-01423-x

**Published:** 2021-05-04

**Authors:** Susan A. Flocke, Elizabeth L. Albert, Steven A. Lewis, Thomas E. Love, Jeanmarie C. Rose, David C. Kaelber, Eileen L. Seeholzer

**Affiliations:** 1 Department of Family Medicine, Oregon Health & Science, University, 3800 SW Sam Jackson Park Rd, Portland, OR 97239 USA; 2 Kaiser Permanente Center for Health Research Northwest, Portland, OR USA; 3 Center for Community Health Integration, Case Western Reserve University, Cleveland, OH USA; 4 Population Health Research Institute, MetroHealth System, OH Cleveland, USA; 5 Center for Health Care Research and Policy, MetroHealth System, OH Cleveland, USA; 6 Department of Population & Quantitative Health Sciences, Case Western Reserve University, Cleveland, OH USA; 7 Department of Medicine, Case Western Reserve University, Cleveland, OH USA; 8 Department of Pediatrics, Case Western Reserve University, Cleveland, OH USA; 9 Center for Clinical Informatics Research and Education, MetroHealth System, Cleveland, OH USA

## Abstract

**Introduction:**

This study examines the uptake of a clinician-focused teachable moment communication process (TMCP) and its impact on patient receipt of tobacco cessation support. The TMCP is a counseling method that uses patient concerns to help clinicians guide behavior change discussions about tobacco. We evaluate the added value of the TMCP training in a health system that implemented an Ask-Advise-Connect (AAC) systems-based approach.

**Methods:**

A stepped wedge cluster randomized trial included eight community health centers. Training involved a web module and onsite skill development with standardized patients and coaching. Main outcome measures included contact and enrollment in cessation services among patients referred for counseling, prescription of cessation medications and quit attempts.

**Results:**

Forty-four of 60 eligible clinicians received the TMCP training. Among TMCP-trained clinicians 68% used a TMCP approach (documented by flowsheet use) one or more times, with the median number of uses being 15 (IQR 2–33). Overall, the TMCP was used in 661 out of 8198 visits by smokers (8%). There was no improvement in any of the tobacco cessation assistance outcomes for the AAC + TMCP vs. the AAC only period. Visits where clinicians used the TMCP approach were associated with increased ordering of tobacco cessation medications, (OR = 2.6; 95% CI = 1.9, 3.5) and providing advice to quit OR 3.2 (95% CI 2.2, 4.7).

**Conclusions:**

Despite high fidelity to the training, uptake of the TMCP approach in routine practice was poor, making it difficult to evaluate the impact on patient outcomes. When the TMCP approach was used, ordering tobacco cessation medications increased.

**Implications:**

Tobacco cessation strategies in primary care have the potential to reach a large portion of the population and deliver advice tailored to the patient. The poor uptake of the approach despite high training fidelity suggests that additional implementation support strategies, are needed to increase sustainable adoption of the TMCP approach.

**Trial Registration:**

clinicaltrials.gov #NCT02764385, registration date 06/05/2016.

## Introduction

The prevalence of smoking in the United States has steadily declined over the past 50 years [1]. However 20.8% of the adult population continues to use tobacco [2] and combustible tobacco use remains one of the major modifiable risk factors for 4 of the 5 leading causes of death [3]. An estimated 60–70% [4] of smokers visit a primary care clinician yearly, underscoring the need for effective smoking cessation interventions within primary care settings.

Systems change interventions in which health information technology is used to identify all smokers and facilitate the documentation and offering of support to smokers into routine care is a promising approach [5, 6]. Systems change approaches have the potential to reach a large segment of the smoking population, and with good design, can be sustainable and add little time to clinical visits [7, 8]. One systems change approach is Ask-Advise-Connect, (AAC) [9, 10] In a manner consistent with the Clinical Practice Guidelines for Treating Tobacco Use and Dependence, AAC engages the medical assistant or nurse that rooms the patient to: **A**sk about tobacco use; for current tobacco users, briefly **A**dvise the patient to quit using tobacco; and offer to **C**onnect patients interested in taking action to quit smoking to cessation counseling support through a quitline referral. For this system, the direct connection to the quitline (QL) is made by clicking a link in the electronic health record (EHR) that securely sends the patient’s name and phone number to the QL staff who contact the patient within 24 h. This is an approach that has been used by others and successfully deployed in multiple primary care settings [9–11]. A challenge with the AAC approach is the modest contact rate among those that are referred to QL [9, 11]. Augmenting the AAC strategy with other personalized approaches may improve patient enrollment in tobacco cessation services and ultimately quitting.

Another promising smoking cessation support approach is advice delivered by clinicians. Research shows that brief advice from a clinician can increase the likelihood of quit attempts and smoking cessation, [12] but routine delivery of brief advice by clinicians is poor. Barriers to brief advice include: lack of time, lack of skills, concern for the clinician-patient relationship, and the perception of insufficient patient motivation [13]. The Teachable Moment Communication Process (TMCP) is a communication strategy, grounded in primary care research and communication theory, designed to engage patients in efficient discussions with their clinicians about quitting smoking. The TMCP operationalizes the process through which teachable moments unfold naturally to provide pragmatic, feasible methods for eliciting a patient’s readiness to quit using tobacco and responding in alignment to that level of readiness. The TMCP enables clinicians to leverage patients’ own concerns and partner in a health behavior change discussion that is integrated into the flow of care and tailored to patients’ priorities and situations. The TMCP has 5 communication elements in which a clinician: 1) identifies a patient’s salient concern, 2) links the concern to tobacco use, 3) provides brief cessation advice, 4) assesses the patient’s readiness to quit, and 5) responds in alignment with the patient’s readiness. In providing brief cessation advice, the TMCP calls on clinicians to convey concern, express optimism and partnership, and make a strong recommendation to quit tobacco [14, 15].

In this study, we examine the combination of the AAC and the TMCP approaches. The AAC intervention is implemented as a systems change designed to be used by the medical assistant or nurse with every patient at every visit. The TMCP is a situation-specific approach intended to be strategically deployed by the clinician when he/she chooses. The AAC and the TMCP interventions have complementary strengths. We hypothesized that the combination of the AAC and the TMCP interventions has the potential to increase the likelihood of patient receipt of tobacco cessation services without diminishing patient report of respect and helpfulness.

## Methods

### Study design

Eight community-based primary care practices in a large safety-net healthcare system in Cleveland Ohio participated in this stepped wedge comparative effectiveness study. The stepped wedge study design was selected for the following reasons: 1) all sites could ultimately receive the intervention which was desirable for the health system; 2) the intensity of the TMCP training was such that the study team could not deliver the intervention at more than one site at a time; 3) the outcomes measured were well suited to assessments across multiple time points. Using a random number list generated by the study analyst, the sites were randomized to separate intervention time points (1-month intervals) in which all clinicians (MD, DO, NPs) who spend 40% or more of the work week providing patient care were scheduled to attend the TMCP training. The health system requested that the intervention be implemented first in one site. The remaining 7 sites followed the randomization procedure. The principal investigator informed each clinical director of the site’s assigned time point and scheduled a training date.

Prior to the baseline period for this study, all eight clinics engaged in an AAC system change initiative to improve the provision of tobacco cessation support [16]. The systems change engaged MAs/RNs to use an electronic health record (EHR) supported AAC strategy. The MA/RN role, which normally included **A**sking about tobacco status, expanded to: 1) Using a brief scripted phrase, to **A**dvise those who use tobacco to quit; 2) Assessing interest in quitting tobacco in the next 30 days; 3) for patients interested in quitting now, asking if they would like assistance to quit from a coach or counselor; and 4) for those choosing assistance, **C**onnecting the patient by an electronic referral (e-referral) sent to either the Ohio Quitline (QL) or to the in-house Freedom from Smoking (FFS) program. Eligibility for free QL services included being aged 18 or older with Medicaid or no insurance, or being pregnant. The QL included up to 5 telephonic counseling sessions and access to web and online chat support. The QL offers nicotine replacement therapy (NRT), if indicated, and with approval from the referring clinician, the NRT is mailed to the patient. Individuals not eligible for the QL were referred to the in-system FFS program – an in-person 8-session group tobacco cessation class offered by the healthcare system. The EHR automatically assessed patient eligibility with payer data and generated the correct referral order in a process that was seamless for both the MA/RN and the patient. The AAC serves as the comparator for the TMCP.

### TMCP Training Implementation

The TMCP intervention included a 50-min web-based module that clinicians completed on office computers, followed by 90 min of skills practices with Standardized Patients (SPs) in the practices’ exam rooms. Trainings were conducted at the practice sites during a clinical work day with cancellation of 2.5 h of clinical activity to accommodate the training.

#### Web Module

The web module consists of: 1) didactic content describing the TMCP rationale and process; 2) actor-portrayed examples of provider-patient interactions in each step of the TMCP; and 3) learning self-assessments. The TMCP training development is described in detail in our previous work [14] and the training content is outlined in Table 1. During the web module, clinicians were asked to interact with the content by repeating certain phrases aloud and thinking about and then saying aloud how they might respond to a patient with a specific level of readiness. Self-assessments (i.e. quizzes) were integrated after each major learning topic. After each question was answered, the clinician was provided with the reason why the response was either correct or incorrect.

**Table 1:** Teachable Moment Communication Process (TMCP) learning module components and objective

Component	Learning objective
**WEB Module – 50 minute interactive learning module**	
1. Call to Action	Alert audience to systems changes at their health system; convey importance of tobacco cessation counseling; introduce the TMCP as skill-building training.
2. Teachable Moment	
A. Rationale	Connect with audience about how difficult it can be to talk about smoking cessation; mention common frustrations and introduce video clip.
B. What not to do	Demonstrate common frustrations and pitfalls of clinicians and patients talking about smoking cessation.
C. Background and overview	Provide specific rationale for TMCP, background research, goals of TMCP; introduce schematic of TMCP; describe what is unique about this approach.
D. Training overview and format	Describe what training consists of and how long it will take.
3. Teachable Moment Communication Process	
A. Overview of 5 elements	Introduce the TMCP 5 elements
B. Salient Concern	*FOR EACH OF 5 ELEMENTS:* Introduce and define it; explain importance of the element; explain how it connects to the element before and after; provide examples to illustrate; present case studies to illustrate variations and flow from one element to the next. Provide several complete case studies to demonstrate the TMCP approach.
C. Making a Link	
D. Brief Intervention using OPEN statements.	
i. Optimism	
ii. Partnership	
iii. Engage to assess readiness	
iv. No more (allow the patient space to respond before moving forward)	
E. Assessing Readiness	
F. Responding in Alignment	
4 TMCP summary and support	
A. Putting it all together	Provide a brief summary of the TMCP and expected outcomes. Describe what to expect when implementing the TMCP with own patients.
B. Coaching session	Introduce and explain Coaching Session
C. Using the EHR Smartphrase	Introduce the Teachable Moment EHR Smartphrase Present the content of the Smartphrase and instruct on how it can be used to document the TMCP
5. Conclusion	Summarize and wrap up
**Skills Practice** – 60 minute active interaction with Standardized Patients and coaches conducted in exam rooms. *Training team consisted of pairs of a coach and a standardized patient. Encounter began with clinician knocking and entering the room and enquiring how can I help you today. SP briefly described reason for visit and revealed a salient concern. Clinicians practiced clarifying salient concern, making a link to tobacco use, expressing concern, assessing readiness and responding in alignment with readiness. Coaches provided feedback, suggestions for improvement and answered questions.*	
6-8 case scenarios	Practice the skills, building self-efficacy with new communication pattern
Coaching Feedback	Fine tune approach, correct misunderstandings, encourage trying variations, reinforce allowing the patient to express themselves and limiting physician actions to match patient readiness.

The training included skills practices for clinicians to learn each TMCP skill through enactment. Skills practices took place in the clinic exam rooms, providing a realistic setting. The clinician participants rotated through 6–8 different scenarios played by SPs. The SPs were actors with prior SP experience and received five hours of training specific to this study. Each SP was trained to act out two unique ‘case scenarios’ that they would routinely play in the skills practices. For each skills practice, the clinician was provided basic SP information, such as age, sex, and smoking history. Skills practices were observed by study team members that served as coaches to provide feedback and additional training in technique. Coaches used a checklist of TMCP skills to insure complete assessment and to guide feedback. Each SP case was also created as a test patient in the EHR so that clinicians could practice using the TMCP document flowsheet with each SP, as described below.

#### Development of document flowsheet

Document flowsheets are EHR tools used to document information in a structured manner. The TMCP flowsheet outlines the steps and phrases for the TMCP for clinicians to use as a guide with patients, eliminating the need to memorize each sequential TMCP step. The flowsheet is also a simple standardized tool to record patient responses with minimal clinician effort. With this tool, both the delivery of TMCP and the recording of patient information are done simply and result in retrievable records for future care.

During the skills training each clinician was shown how to use the TMCP flowsheet and had it added to his/her EHR tool shortcuts. Clinicians were asked to use the flowsheet during the skills practice scenarios to gain experience and comfort with its features. As needed, coaches could address any difficulty clinicians had using the flowsheet during the feedback period after a skills practice. An EHR tobacco cessation order set could be used by providers to order tobacco cessation medication and to order QL or FFS classes and was also reviewed during skills practice.

#### Debriefing about the training and follow-up

Following the completion of the web-module and the skills practices, the providers re-grouped with the research team to debrief about their experiences. Feedback on format, length of training, processes, content etc. were discussed as well as the perceived value overall. Approximately one month after the TMCP training participating clinicians were sent follow-up messages through the EHR that 1) offered reminders about the TMCP, 2) encouraged clinicians to use the TMCP, and 3) provided instruction for using the tobacco cessation flowsheet. Clinicians were invited to respond to messages with questions and or feedback.

### Study sample

**Fig. 1:**
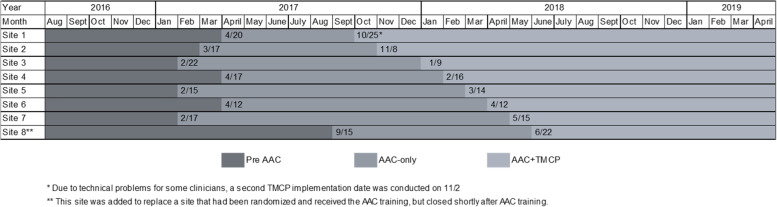
Schematic of the timing of the AAC and the TMCP interventions at each of the 8 study sites

The study sample consists of eight sites, 60 clinicians from those sites and all adult patient visits during the study period. Figure 1 shows the timing of the training and implementation of the TMCP interventions at each of the eight study sites. Study data collection began August 2016 and concluded April 2019. One of the original eight clinics that received the AAC training closed shortly afterwards. A replacement site (#8) was identified from a similar neighborhood, and the original clinic was excluded from all analyses. The replacement site received the AAC training and was assigned to receive the TMCP intervention training 1 month after the TMCP training implementation was conducted at the last clinic that had been randomized. This approach allowed sufficient time for data collection and assessment between the implementation of the AAC and the implementation of the TMCP intervention.

### Measures

Clinician characteristics gathered from the health system included: sex, degree (MD, DO, NP), specialty and years since last clinical training. Patient characteristics including sex, race, ethnicity, insurance type and smoking status, were drawn from the EHR.

TMCP-specific variables include training participation, online learning module completion and quiz score. Use of the TMCP flowsheet by clinicians during visits was measured for 6-months after training to indicate use of the TMCP. Clinician’s documentation of any portion of the TMCP flowsheet was counted as ‘use’.

Outcome measures include contact by the QL or FFS among those patients that accepted a referral and provision of brief advice. Secondary outcomes include medication orders (i.e. varenicline, bupropion or nicotine replacement therapy) enrollment among those referred to cessation support (QL or FFS), and quit attempts. For this study, a quit attempt is defined as a change in smoking status from current smoker to former smoker and documentation of a quit date in the EHR. Quit attempts are evaluated for all smokers.

We report the rates of Ask, Advise, Assessment of readiness to quit and acceptance of a referral (Connect) to tobacco cessation assistance for the AAC intervention (AAC only) period and the time period after the TMCP intervention (AAC + TMCP). Each of these indicators is measured from discrete fields in the EHR. As shown in Fig. 1, the timing of the AAC and the TMCP intervention implementation and the time periods between the two interventions varied.

We assessed patients’ experiences with the clinic’s processes. The survey samples included a minimum of 17 smokers from each of the eight sites for the AAC only and the AAC + TMCP study periods. Eligibility for the survey included patients 18 years of age or older who attended a primary care visit during a specified period. For those individuals who had an email address as part of his / her record, an email invitation and a link to an online survey was sent. For those with no email, a letter was followed by a phone call to invite completing the survey over the phone. The survey included 11 items about being treated with respect, feeling listened to, and whether the advice was helpful. The items were adapted from the Communication Assessment Tool (CAT) [17] to be specific to discussions about smoking. Responses were measured using a 5- point Likert scale rating from excellent to poor.

### Statistical analyses

We describe clinician characteristics and patient visits during the study period, and participation in the training and uptake of the TMCP as documented by flowsheet usage. Analyses compared rates on outcomes for the AAC + TMCP intervention to AAC only period using generalized linear modeling along with generalized estimating equations (GEE) methods.

GEE was used to account for the lack of independent observations due to the clustering of patients within clinics. It uses robust variance estimation, rather than traditional maximum likelihood estimation techniques to adjust standard errors, and thereby produces more accurate model estimates.

Specifically, we modeled the log odds of a particular outcome of interest for a particular patient as a function of intervention status (AAC only vs. TMCP + AAC) at each time point, incorporating random practice site and clinician effects. Three analyses were conducted: 1) intent to treat, 2) per protocol (i.e. clinicians that participated in the TM training), and 3) per documented use of the TMCP approach in a visit. We report rates of performance, odds ratios (OR) and 95% confidence intervals (95% CI). We used 10% as a minimally important difference. Data analyses were performed April 2019—August 2020. Because this was an educational intervention for clinicians, all of whom ultimately received the training, neither the clinicians nor the study team could be blinded to the group assignment; this design also made it impossible to blind the analyst to group assignment.

## Results

Of the 60 clinicians at the 8 study sites, 44 received the TMCP training. (See Table 2) Those that received training were similar in years since last clinical degree, degree type and internal vs family medicine to those that did not. Women were underrepresented among TMCP-trained clinicians compared to non-trained clinicians. Reasons for not receiving the training were related to time-off due to part-time status, leave, or vacation and patient care or administrative duties at a different location on training day. Among TMCP training participants, 57% were family medicine and 43% were internal medicine specialty; 68% were MD, 11% DO, and 21% were certified nurse practitioners. The average years since last clinical degree was 16.3 years (SD = 11.0).

**Table 2:** Clinician characteristics

Characteristics	Received TMCP Training* n = 44	No TMCP Training *n* = 16	P
Gender			
Male	19 (43%)	1 (6%)	0.001^**^
Female	25 (57%)	15 (94%)	
Training			
MD	30 (68%)	7 (44%)	0.09
DO	5 (11%)	1 (6%)	
NP	9 (21%)	7 (44%)	
PA	0 (0%)	1 (6%)	
Specialty			
Family Medicine	25 (57%)	7 (44%)	0.37
Internal Medicine	19 (43%)	9 (56%)	
Years since last clinical training Mean, (std dev)	16.3 (11.0)	11.4 (9.4)	0.12

* All clinicians that were present at the practice on the assigned training day participated in the training. Reasons for not participating: maternity leave, patient care at a different site on that day, planned time off

Boldface indicates statistical significance (^**^p < 0.01)

Table 3 describes characteristics of patient visits across the study time periods. There were no substantial differences between the AAC-only and the AAC + TMCP time periods in the characteristics of the patients making visits. Overall, 62.6% of visits were by females, 51.3% were by whites, 43.2% by blacks and 15.1% were by Hispanics (white or black). Most had government insurance, Medicaid (36.6%) or Medicare (28.6%); 28.2% were commercially insured. About 23% of visits were by individuals who smoke and 25% by former smokers. Although the volume of patients seen at each of the sites varied, overall volume was relatively stable across the time periods.

**Table 3:** Characteristics of patient visits for the AAC only and AAC + TMCP periods*

		Overall *N* = 119,274	AAC only *N* = 77,500	AAC + TMCP *N* = 41,774
**Description**	**Category**	**N (%)**	**N (%)**	**N (%)**
Gender	Male	44,434 (37.3%)	28,965 (37.4%)	15,469 (37.0%)
	Female	74,840 (62.7%)	48,535 (62.6%)	26,305 (63.0%)
Age in years	18–34	20,112 (16.9%)	13,048 (16.8%)	7,064 (16.9%)
	35–64	72,788 (61.0%)	47,683 (61.5%)	25,105 (60.1%)
	65 +	26,374 (22.1%)	16,769 (21.6%)	9,605 (23.0%)
Race	White	54,226 (51.3%)	35,017 (51.7%)	19,209 (50.6%)
	African American	45,711 (43.2%)	29,739 (43.9%)	15,972 (42.1%)
	Other	5,767 (5.5%)	2,979 (4.4%)	2,788 (7.3%)
Hispanic	Non-Hispanic	98,711 (84.9%)	63,928 (84.6%)	34,783 (85.4%)
	Hispanic	17,587 (15.1%)	11,627 (15.4%)	5,960 (14.6%)
Primary Insurance Class	Commercial	32,979 (28.2%)	21,377 (28.2%)	11,602 (28.2%)
	Medicaid	42,792 (36.6%)	27,987 (36.9%)	14,805 (36.0%)
	Medicare	33,440 (28.6%)	21,438 (28.3%)	12,002 (29.2%)
	Self-Pay	7,647 (6.5%)	4,945 (6.5%)	2,702 (6.6%)
	Other	123 (0.1%)	85 (0.1%)	38 (0.1%)
Smoking Status	Current Smoker	26,903 (22.6%)	17,408 (22.5%)	9,495 (22.7%)
	Former Smoker	29,411 (24.7%)	18,961 (24.5%)	10,450 (25.0%)
	Never Smoked	43,748 (36.7%)	28,177 (36.4%)	15,571 (37.3%)
	Not Assessed	19,212 (16.1%)	12,954 (16.7%)	6,258 (15.0%)
Readiness to Quit Assessed	No	8,008 (37.2%)	4,494 (33.7%)	3,514 (42.9%)
	Yes	13,532 (62.8%)	8,848 (66.3%)	4,684 (57.1%)
Ready to Quit	No	9,769 (72.2%)	6,158 (69.6%)	3,611 (77.1%)
	Yes	3,763 (27.8%)	2,690 (30.4%)	1,073 (22.9%)
Health Center	1	7,609 (6.4%)	4,785 (6.2%)	2,824 (6.8%)
	2	22,111 (18.5%)	14,703 (19.0%)	7,408 (17.7%)
	3	10,432 (8.7%)	4,797 (6.2%)	5,635 (13.5%)
	4	14,394 (12.1%)	9,441 (12.2%)	4,953 (11.9%)
	5	20,036 (16.8%)	13,877 (17.9%)	6,159 (14.7%)
	6	22,159 (18.6%)	13,975 (18.0%)	8,184 (19.6%)
	7	18,341 (15.4%)	13,483 (17.4%)	4,858 (11.6%)
	8	4,192 (3.5%)	2,439 (3.1%)	1,753 (4.2%)

^*^ the data in this table are limited to the visits for the per protocol analysis

The TMCP training web module was fully completed by all but one clinician, who completed 80%. The average score on the module quiz was 80% correct (10 individuals scored ≥ 90%). Feedback from the clinicians about the web module, skills practice section, and place and timing of the training was positive. During 6 months after training, 68% of 44 trained clinicians used TMCP document flowsheet at least once and the median number of uses was 15 (IQR 2–33). Seventeen clinicians (39%) used the document flowsheet > 10 times and four used it > 50 times. In all, a document flowsheet was used in 660 of 8199 visits (8%) by smokers during the evaluation period.

The intent to treat analyses included all 60 clinicians regardless of whether they received training; there were no substantial increases in any of the process or outcome indicators when we compare the AAC only period to the AAC + TMCP period (data not presented). The data in Table 4, which show the per-protocol analyses, are limited to the visits to one of the 8 sites in each time period and the 44 clinicians that participated in the TMCP training. Comparing the AAC only to the AAC + TMCP, some tobacco assistance behaviors increased: Asking about tobacco status (which was done by the MA), and provision of advice (which was done by the MA and/or the clinician). Other behaviors decreased, including assessing patient readiness to quit and patient acceptance of external referrals. There were no meaningful increases in patient outcomes (i.e. contact or enrollment rate for those referred or quit attempts). Ordering of medications decreased from 14.9% for the AAC only to 12.4% for the AAC + TMCP period. For visits in which clinicians documented use of the TMCP approach (last column of Table 4), improved outcomes were observed for advising to quit OR 3.2 (95% CI 2.2, 4.7) and ordering tobacco cessation medications OR 2.6 (95% CI 1.9–3.6). Data are presented for the main outcomes of contact and enrollment in QL counseling, however, the number of cases are too few to meaningfully interpret.

**Table 4:** Effect of AAC + TMCP on provision of tobacco cessation support

	AAC only		AAC + TMCP			AAC + TMCP—among clinicians who used the Document FlowSheet		
	N	%	N	%	OR (95% CI)^a^	N	% OR (95% CI)^b^	
*Total VISITS*	*77,500*		*41,774*			*661*		
Asked	38,687	49.9%	26,452	63.3%	1.73 (1.70, 1.77)	492	74.4%	1.89 (1.62, 2.20)
*Visits by smokers*	*13,342*		*8198*			*466*		
Advised	11,323	84.9%	7466	91.1%	1.97 (1.81, 2.14)	443	95.1%	3.21 (2.19, 4.72)
Assessed Readiness	8848	66.3%	4684	57.1%	0.66 (0.62, 0.69)	347	74.5%	1.24 (1.00, 1.54)
*Ready to quit*	*2690*		*1073*			*93*		
Accepted Any Referral	1176	43.7%	369	34.4%	0.66 (0.57, 0.77)	43	46.2%	1.00 (0.66, 1.51)
Accepted QL Referral	827	30.7%	252	23.5%	0.68 (0.58, 0.80)	22	23.7%	0.71 (0.45, 1.14)
Accepted FFS Referral	349	13.0%	117	10.9%	0.79 (0.64, 0.98)	21	22.6%	1.45 (0.85, 2.49)
PATIENTS *Ready to quit*	*2239*		*1001*			*93*		
Accepted Referral	1089	48.6%	357	35.7%	0.59 (0.47, 0.74)	43	46.2%	0.80 (0.50, 1.29)
Accepted QL Referral	765	34.2%	247	24.7%	0.64 (0.50, 0.82)	22	23.7%	0.58 (0.38, 0.90)
*QL Referrals Received*	*649*		*200*			*15*		
QL Contact	211	32.5%	44	22.0%	0.58 (0.36, 0.95)	3	20.0%	0.53 (0.18, 1.55)
QL Enrollment	158	74.9%	29	65.9%	0.65 (0.35, 1.23)		33.3%	0.15 (0.01, 1.96)
Accepted FFS Referral	328	14.7%	112	11.2%	0.74 (0.58, 0.95)	21	22.6%	1.42 (0.86, 2.34)
*FFS Referrals Received*	*276*		*106*			*21*		
FFS Contact	75	*27.2%*	23	*21.7%*	*0.82 (0.53, 1.26)*	5	23.8%	1.00 (0.46, 2.18)
FFS Enrollment	50	66.7%	18	78.3%	1.74 (0.47, 6.40)	3	60.0%	0.79 (0.12, 5.18)
Medications	1126	14.9%	718	12.4%	0.81 (0.74, 0.88)	139	3.7%	2.61 (1.92, 3.55)
*Smokers with two or more visits in period*	*2132*		*1996*			*216*		
Quit attempts ^c^	44	2.1%	45	2.3%	1.09 (0.72, 1.67)	8	3.7%	1.95 (0.86, 4.45)

^a^ Comparison is post-TMCP to post-AAC period among clinicians who received training

^b^ Comparison is post-TMCP for visits in which a document flowsheet was used compared to visits during the post-AAC period to clinicians that ever used the doc flowsheet

^c^ Quit attempts are defined based on 2 or more visits in the time-period, a change in smoking status from current smoker to former smoker and documentation of a quit date

A total of 169 patients completed the survey in the AAC only and 164 in the AAC + TMCP period; the response rate was 18%. The patient report of the experience for each item and across time periods were in the very good to excellent range (i.e. scores 4–5). Using ANOVA and planned paired (Tukey HSD) comparisons, no differences were statistically significant or clinically meaningful. This indicates that ratings of helpfulness and respect during tobacco discussions were rated similarly for the AAC only and the AAC + TMPC time frames.

## Discussion

This is the first study to engage clinicians in the TMCP as part of a systems change intervention. Prior work engaged clinicians as study volunteers [14, 18]. The training for this study was conducted at the practice site, during a clinical work day in which the health care system allowed cancellation of 2.5 h of clinical care. This resulted in a high attendance at the trainings. Feedback from the clinicians about the web module, skills practice section, and place and timing of the training was positive and the TMCP approach and training were viewed as both important and pragmatic. Clinicians’ understanding of the TMCP concepts is indicated by high scores on the quizzes embedded in the training and demonstrated through competency in adequately completing the communication process during skills practices with SPs. This study also developed and deployed a flowsheet, which is a common EHR tool, to both cue and document use of the TMCP during patient care. Use of the flowsheet served as an indicator that the TMCP was used.

Despite a robust method for training and development of support tools, the TMCP approach was used in only 8% of visits with smokers, and TMCP training had no substantial impact on the indicators examined in this study. Like other EHR tool implementation efforts, our findings show substantial variability in the adoption and use of this EHR tool despite specific training and technical assistance [19]. However, among visits in which the approach was documented as *used*, there was a large increase in provision of advice from 84.9 to 95.1% and in ordering of tobacco cessation medications from 14.9 to 31.6%. The impact on increasing tobacco cessation medication orders is important in that pharmacotherapy, even in the absence of cessation counseling, has been shown to increase the likelihood of a quit attempt by two to three-fold [20, 21].

Unlike the AAC approach, which was systematically built into the work flow of every visit, the TMCP was designed to be used at the clinician’s discretion when a salient patient concern made discussion of tobacco cessation relevant to the visit. It was not expected that clinicians would deploy the TMCP approach in every visit, however, salient concerns relevant to tobacco cessation discussions commonly arise during routine primary care visits [15]. It is reasonable to expect the TMCP to be used in 25% or more of visits with patients using tobacco [22]. The overall low uptake of the TMCP approach in routine care limits the evaluation of its impact on patient outcomes. Other strategies that might be considered in future research to improve clinician uptake include conducting audit and feedback on performance [23] and provision of booster training.

This study anticipated positive synergy between the AAC and TMCP interventions. Offered at all visits, AAC reaches a large number of patients. For patients motivated to quit in the next 30 days, AAC offers immediate, effective assistance via proactive outreach from the state QL. For all patients who smoke, regardless of readiness to quit now, the TMCP enables a supportive and partnership-oriented health behavior change discussion. Ideally, after meeting with the MA/RN, the clinician could review the outcome of the AAC interaction. However, during the study we discovered that within the clinicians’ view of the EHR for a visit, clinicians could not easily see that information. This communication gap largely prevented patients who had agreed to the MA/RN’s tobacco cessation counseling referral from receiving responsive clinician encouragement and the offer of medication support for increased quitting success. It is possible that improved connection of AAC to clinician visits would have increased clinician TMCP use and improved patient outcomes.

Another opportunity for increased synergy between the two approaches is based on the recognition that smoking cessation is typically a process wherein smokers try to quit and then lapse several times before succeeding long-term. Recent reports indicate that the average smoker may make more than thirty quit attempts before finally quitting [24]. In this study the QL was able to contact just 32.5% of patients who agreed to a referral. However, research shows that patients desire continued follow-up discussion with their primary care clinician regarding smoking cessation [25]. An important opportunity to use the TMCP is to engage patients in follow-up discussions about smoking cessation progress, help address barriers, promote medications to support success, and encourage the value of continued attempts after a lapse.

The low adoption of the TMCP approach limits the ability to examine its impact in concert with the AAC. The AAC shows good reach and impact in this initiative and elsewhere, [9, 16] but an important role for clinicians in providing tobacco cessation support remains. Specifically, the influence of a quit message from a clinician reinforces the importance and priority of tobacco cessation, especially in the context of patient symptoms or chronic conditions. Future work should focus on system support to integrate these interventions and increase sustainable adoption of the TMCP in order to assess its impact in combination with AAC.

### Study limitations

The study sample was drawn from a health center serving patients who have predominately very low income, thus socio-economic barriers to engaging with the QL (e.g. perceived lack of time, telephone access) were prevalent. The smoking rate in the practices is about 22%, and it is unknown how this or other patient characteristics might impact uptake and use of the TMCP approach. Uptake of the TMCP approach was assessed by use of the flowsheet, which misses instances where the approach was used, but not documented. While no differences were observed in patient report of being treated with respect and the advice being helpful between the AAC and the AAC + TMCP periods, re-evaluation of the patient perspective after a more robust implementation of the TMCP approach is warranted. A quit attempt is calculated using documentation of a quit date in the EHR. However, quit attempts may have been made by patients without supportive documentation. Therefore, our quit attempt estimate is a lower bound for the actual quit attempt rate. Finally, the observed variation in TMCP uptake by clinicians did not include clinician level variables that might explain some of this variation. Despite these limitations, the findings are relevant and applicable to other primary care settings.

## Conclusions

In a health system using an AAC systems approach, the additional use of the TMCP by clinicians improved documentation of provision of advice and ordering tobacco cessation medications. No other improvement in outcomes was observed. Despite high fidelity to the training, overall clinician uptake of the TMCP approach in routine practice was low, making it difficult to evaluate its impact on patient outcomes. It is likely that with improved integration of the AAC and TMCP through EHR tools and team processes, the combined interventions will have a greater impact on patient tobacco cessation outcomes.

## Data Availability

A limited dataset may be made available upon request specifying intent of use. No individual identifiable data will be shared.
